# Heart rate variability may index emotion dysregulation in alcohol-related intimate partner violence

**DOI:** 10.3389/fpsyt.2023.1017306

**Published:** 2023-02-28

**Authors:** Brandi C. Fink, Eric D. Claus, James F. Cavanagh, Derek A. Hamilton, Judith N. Biesen

**Affiliations:** ^1^The Department of Psychiatry and Behavioral Sciences, The University of New Mexico, Albuquerque, NM, United States; ^2^The Mind Research Network, Albuquerque, NM, United States; ^3^Department of Psychology, The University of New Mexico, Albuquerque, NM, United States; ^4^Department of Mental Health Law and Policy, University of South Florida, Tampa, FL, United States

**Keywords:** intimate partner violence, alcohol, heart rate variability, emotion regulation, couple conflict

## Abstract

**Introduction:**

Intimate partner violence is a serious public health problem that costs the United States more than $4.1 billion in direct medical and mental health costs alone. Furthermore, alcohol use contributes to more frequent and more severe intimate partner violence incidents. Compounding this problem is treatments for intimate partner violence have largely been socially informed and demonstrate poor efficacy. We argue that improvements in intimate partner treatment will be gained through systematic scientific study of mechanisms through which alcohol is related to intimate partner violence. We hypothesize that poor emotional and behavioral regulation as indexed by the respiratory sinus arrythymia measure of heart rate variability is a key mechanism between alcohol use and intimate partner violence.

**Method:**

The present study is a placebo-controlled alcohol administration study with an emotion-regulation task that investigated heart rate variability in distressed violent and distressed nonviolent partners.

**Results:**

We found a main effect for alcohol on heart rate variability. We also found a four-way interaction whereby distressed violent partners exhibited significant reductions in heart rate variability when acutely intoxicated and attempting to not respond to their partners evocative stimuli.

**Discussion:**

These findings suggest that distressed violent partners may adopt maladaptive emotion regulation strategies such as rumination and suppression when intoxicated and attempting to not respond to partner conflict. Such strategies of emotion regulation have been shown to have many deleterious emotional, cognitive and social consequences for individuals who adopt them, possibly including intimate partner violence. These findings also highlight an important novel treatment target for intimate partner violence and suggest that novel treatments should focus on teaching effective conflict resolution and emotion-regulation strategies that may be augmented by biobehavioral treatments such as heart rate variability biofeedback.

## Introduction

Intimate partner violence (IPV) is a significant public health problem costing more than $5.8 billion annually with more than $4.1 billion in direct medical and mental health services alone. There are approximately 22.4 million physical assaults committed by a current or former intimate partner per year against an estimated 10 million Americans (1, 2) and an increasing number of homicides by intimate partners (3). National surveys reveal that nearly one third of couples will experience physical aggression at some point in their relationship and 35% of couples will experience IPV in any given year (4). Compounding the issue of IPV is alcohol use. Alcohol use has been found to be present in most instances of IPV (57 to 70% of IPV incidents), and more severe IPV incidents occur during heavier drinking episodes [e.g., binge drinking; (5–10)]. Although the association between alcohol use and intimate partner violence is well established, the mechanisms of this association are poorly understood, which has stymied the development of treatments that effectively engage these mechanisms to produce appreciable decreases in IPV (11–16).

### Distressed violent couples’ behavior and physiological over-arousal

Distressed violent couples engage in several unique dyadic behavioral and affective patterns that escalate conflict and physiological arousal more than distressed nonviolent couples. Distressed violent couples are more likely to engage in *negative reciprocity,* which is the tendency to continue or escalate negative and evocative behavior once it begins (17). They also display abnormal *demand-withdraw patterns* (18–20). Individuals exhibiting demanding behavior generally want more intimacy or closeness in an interaction and individuals displaying withdrawing behavior generally want greater autonomy or separateness. If this demand-withdraw pattern is present in a couple interaction, one partner typically exhibits demanding behavior while the other partner exhibits withdrawing behavior. In distressed violent couples, however, partners alternate these behavioral patterns. For example, expressions of desires for assistance, closeness or intimacy by each partner are met by withdrawal by the other partner; a dynamic that lays the foundation for high conflict, power struggles, clinging, and hypervigilant responses; all experiences reported by distressed violent couples (21, 22).

Distressed violent couples are further distinguished from distressed nonviolent couples in both partners’ propensity to express blends and higher levels of negative affect, such as contempt and belligerence, which escalate conflict beyond that seen in distressed nonviolent couples (23). In fact, distressed violent couples become more psychologically abusive, emotionally aggressive, and increasingly physiologically aroused as their conflict continues because of these patterns (18, 24–26). Distressed violent couples also have difficulty disengaging from conflict once it begins without escalating to physical aggression due to their inability to regulate affect and behavior when in a highly aroused state (24–28). Furthermore, partners in relationships whose conflict chronically generate such arousal become hypervigilant to potentially threatening and escalating interactions and are more likely to misattribute threat potential to relatively neutral or positive acts (29) suggesting a sensitization process whereby repeated exposures to aversive dyadic interactions result in a progressive amplification of the arousal response to the partner’s behavior.

#### Sympathetic dominance

Research suggests that the unique dyadic patterns seen in distressed violent couples may be moderated by low heart rate variability and a shift to dominance of the sympathetic branch of the autonomic nervous system. For example, low heart rate variability has been associated with suppressed anger and social isolation (30), aversive reactions to harmless, nonthreatening stimuli (31), and more extreme evaluations of blame in anger-inducing situations (32). Seminal research on marital interactions of nonviolent couples (33) found that blends of high levels of negative affect (contempt, belligerence, criticism, defensiveness and stonewalling) increased activation of the sympathetic branch of the autonomic nervous system and that this shift to sympathetic dominance was associated with a loss of affect and behavioral regulation in these marital interactions.

### Alcohol’s effect on affect regulation and aggression

In addition to the physiological changes caused by conflict and emotional stress, low to moderate alcohol exposure also leads to decreases in heart rate variability measures of parasympathetically mediated cardiac activity (34–37) and a shift toward sympathetic dominance (38). Although alcohol is classified as a pharmacological depressant, during the absorption phase (ascending limb of intoxication), and at peak Blood Alcohol Concentration (BAC), alcohol is actually neuropsychophysiologically arousing (39–41). Like experimental studies of arousal and aggression, experimental studies of alcohol and aggression find that alcohol is associated with aggressive behavior only under conditions of provocation and frustration [see Exum (42) for review]. Furthermore, experimental manipulation of alcohol limb effects (i.e., ascending vs. descending limb) have provided evidence of increased aggressive tendencies on the ascending limb compared to the descending limb, thus providing further evidence of the physiological influence of alcohol playing a facilitative role in IPV due to the disruption in normal physiological functioning (43).

#### Present study

The present study integrates and extends the findings of previous research examining the physiological changes in distressed nonviolent couples and alcohol-related aggression to understanding a potential mechanism of alcohol-related intimate partner violence. In the present study distressed violent and distressed nonviolent partners were matched on sex, age and relationship distress and participated in a placebo-controlled alcohol administration study with an emotion-regulation task during which electrocardiogram measures of heart rate variability (HRV) were recorded. The HRV measure of interest in the present study is respiratory sinus arrythmia (RSA) as it is an index parasympathetic activation and partners’ abilities to respond adaptively to interoceptive (strong affect blends) and exteroceptive (evocative partner behavior) stimuli. We made four hypotheses in this study. First, we hypothesized that alcohol would reduce respiratory sinus arrythmia in in partners consistent with previous literature. Second, we hypothesized that distressed violent partners would exhibit lower respiratory sinus arrythmia when intoxicated than distressed nonviolent partners. Third, distressed violent partners would exhibit lower respiratory sinus arrythmia when intoxicated and attempting to regulate emotion than distressed nonviolent partners. Lastly, we hypothesized, compared to distressed nonviolent partners, acute alcohol intoxication would produce lower respiratory sinus arrythmia, in distressed violent partners when in a highly arousing condition of being asked to view evocative partner stimuli while being asked to feel the emotions associated with those evocative stimuli.

## Methods

### Participants

Data from partners in the present study were drawn from a parent study investigating over-arousal as a mechanism between alcohol use and partner violence (AA022367). Participants were recruited from the community through radio, television and newspaper advertisements seeking opposite sex couples who were experiencing conflict in their relationships and who drank alcohol to participate in a research study examining emotions and cognitions in conflict. Eligibility screening occurred at the couple level. Eligible couples were (1) English speaking, (2) heterosexual, (3) age 21–45-years-old, (4) in a distressed relationship, (5) had two binge drinking episodes in the previous 30 days (to qualify for an alcohol-administration study), (6) were married or cohabitating at least 6 months, (7) showed no signs of physical aggression outside of the intimate partner relationship, and (8) provided a breath alcohol level of 0.0 g% at all visits. Distressed violent partners exhibited at least mild physical aggression (e.g., pushed or shoved partner, twisted partner’s arm or hair) in the previous 6 months, whereas distressed nonviolent partners exhibited only relationship distress. The age range of participants reflects the legal drinking age and a range that reduces heterogeneity due to age-related changes in the physiological measures collected. Participants were excluded if they (1) were currently separated, (2) had an order of protection in place, (3) were facing violence-related criminal charges, (4) were currently in a domestic violence shelter, (5) presented with evidence of psychosis or severe personality disturbance, (6) were pregnant (female participants were pregnancy tested at all experimental sessions), (7) were taking a medication contraindicated for use with alcohol, (8) were currently taking insulin or oral hypoglycemic medication, (9) had an alcohol use disorder identification test score > 19 and/or indicating alcohol dependence symptoms, (10) reported illicit drug use (except marijuana) and (11) provided a positive urinalysis for opioid or illicit drug use at the stimuli acquisition session.

Participants in the present analyses were 26 distressed violent (18 female, 8 males) and 16 distressed nonviolent partners (7 females, 9 males). The mean age of the sample was 32 (SD 4.8 years, range 23–40 years). Fifty-one percent of participants were Hispanic, 27% White, Non-Hispanic, 10% African American, 7% Native American, and 5% self-identified as other race/ethnicity (e.g., Asian/Pacific Islander, Mexican-American).

### Ethical considerations

The study was approved and overseen by the Human Research Review Committee of the academic health center in the southwest United States where the study was conducted. There were protections in place both for IPV and for alcohol consumption. Protections for IPV: both partners completed a mood survey at the conclusion of the stimuli acquisition session, and at the conclusion of the experimental session by the participating partner. Participants could not rate feeling worse than ‘slightly negative’ on a scale ranging from ‘very negative’ to ‘very positive’ and be dismissed from the study session. If one or both partners rated feeling worse than ‘slightly negative,’ they were interviewed by the PI, a licensed clinical psychologist, who used interviewing techniques to de-escalate the partner(s). Each partner was also phoned 24 h after each study session, and 1 week after completion of the experimental sessions to ensure that study procedures did not contribute to a violent argument between partners. Assurance was sought from each partner that he/she was alone when responding to these questions. Each partner was also individually provided with referral materials to therapy and legal resources.

Protections for the consumption of alcohol included participants being required to have reported at least two binge drinking episodes in the previous month (>4 drinks for males, >3 drinks for females) to ensure that participants were not dosed at a level of alcohol that they were unaccustomed to achieving on their own. Pregnancy testing was completed for all female participants before the placebo and the alcohol conditions. During detoxification, participants were breathalyzed every 15 min and required to remain in the laboratory until two consecutive breath alcohol concentration (BAC) readings of 0.03% or below were achieved, as recommended by the NIH National Institute on Alcohol Abuse and Alcoholism (NIAAA) guidelines for the safe release of participants.^1^

### Materials

#### Psychosis and severe personality disturbance

To exclude potential participants with psychosis or anti-social personality disorder, both partners of each couple completed the Structured Clinical Interview for DSM-IV Axis II Personality Disorders (44). Because this study was investigating alcohol-related intimate partner violence in family-only violent couples, participants were also screened and excluded if they reported facing violence-related criminal charges as this is indicative of individuals who are violent outside of their intimate relationships.

#### Alcohol use

For the purposes of assessing alcohol use for meeting exclusion criteria at screening, both partners completed an Alcohol Use Disorders Identification Test [AUDIT; (45)]. Participants who scored a 10 or above (indicating hazardous drinking) were provided with a brief intervention for alcohol use, provided with an active referral to treatment and excluded from the study.

To ensure that participants consumed sufficient amounts of alcohol to ensure that alcohol administration procedures would not result in alcohol levels higher than what participants routinely achieved on their own, a Timeline Follow-Back (46) was completed. Partners who were selected for the experimental conditions reported two binge (per sitting, three or more standard drinks for females, or four or more standard drinks for males) alcohol drinking episodes in the previous 30-days. Secondly, consistent with assessing violence in the past 6 months in couples, drinking was assessed. There were no significant differences between distressed violent (M = 185.56, SD = 221.27) and distressed nonviolent partners (M = 222.57, SD = 308.65) in standard drinks consumed in the previous 6 months (*t* = −0.439, *p* = 0.133).

#### Relationship distress

Relationship distress was determined using the total score of Dyadic Adjustment Scale [DAS; (47)]. The DAS is a 32-item measure of relationship quality that is divided into four subscales: dyadic consensus, dyadic satisfaction, dyadic cohesion and dyadic affection. Total scores of 97 or less reflect relationship distress. For partners of a couple who did not both have DAS scores less than 97, the couple was considered distressed if their averaged DAS score was 97 or less. The mean total score in the current study was 94.27 (SD = 20.26, range 52.00–124.00). There were no significant differences in relationship distress between distressed violent and distressed nonviolent partners (*t* = −1.567, *p* = 0.126). In the current study, Cronbach alpha for the total scale was 0.92.

#### Intimate partner violence

For the purposes of partner classification, IPV was determined using the revised conflict tactics scale [CTS2; (48)]. The CTS2 is 39-item paired self-report and partner report scale developed to assess the use of tactics by partners in resolving conflict. The CTS2 is comprised of five subscales that include: negotiation, psychological aggression, physical assault, sexual coercion and injury. In using the CTS2 to classify distressed violent and distressed nonviolent partners, the physical assault subscale was consulted, and a couple was classified as distressed if a partner self-reported the use of physical aggression toward his or her intimate partner. The Cronbach alpha for the total scale in this sample was 0.90, and 0.63 for the physical assault subscale.

#### Partner stimuli for emotion-regulation task

Video clips of partner stimuli selected for use in the emotion-regulation task were obtained from a researcher facilitated discussion of a disagreement that occurred in the initial couple session of the study. Using the Couple Problem Inventory [CPI; (49)], partners identified areas of disagreement that were most significant for them. The couple was then asked to discuss the area of disagreement for 15 min and attempt to research a resolution. A video camera was trained on the head and shoulders of each partner and videos were later coded using the Specific Affect Coding System (50). Twenty-five video clips that were approximately four to 8 s in length and of displays of contempt, belligerence, criticism, defensiveness and stonewalling were selected for presentation during the evocative condition of the emotion-regulation task. Twenty-five video clips of neutral behavior were selected for presentation during the neutral control condition of the emotion-regulation task.

#### Anger expression

Anger expression was measured using the STAXI-2 (51). The STAXI-2 is a self-report questionnaire that measures the experience, expression, and control of anger in both research and clinical samples. The STAXI-2 is comprised of six scales (state anger, trait anger, anger expression-out, anger expression-in, anger control-out and anger control-in). Responses are made on a likert-type scale ranging from 1 (almost never) to 4 (almost always). Trait anger indexes frequent angry feelings and feeling of being treated unfairly. Anger expression-out indexes anger expressed in verbally or physically aggressive behavior directed at others or objects. Anger expression-in indexes the suppression of frequent intense angry feelings. Anger control-out is an index of effort expended in the monitoring and prevention of outward experiences and expressions of anger. Anger control-in indexes effort expended in calming down, and reducing angry feelings immediately, which reduces awareness of when assertive behavior is needed in facilitating constructive resolutions to conflict situations. Mean scale responses from the trait anger, anger expression-out, anger expression-in, anger control-out and anger control-in were used for analysis. In the current study, the Cronbach alpha for the trait anger scale was 0.85, anger expression-out was 0.63, anger expression-in was 0.81, anger control-out was 0.82 and anger control-in was 0.86. As reported in our previous work (52), there were no significant differences in anger experiences between partner types.

### Procedure

The present study was a counter-balanced placebo-controlled alcohol administration study that consisted of three sessions; an initial stimuli acquisition session that involved both partners, and two experimental sessions that involved only one partner. Data presented here were drawn from the experimental sessions. Distressed violent partners were pseudo-randomly selected for participation in the experimental sessions. If gender symmetry in the use of physical aggression was reported by a couple, a partner was randomly selected for participation. If the couple was asymmetrical in their self-reported use of physical aggression, the partner self-reporting the greatest use of physical aggression was invited to participate. Distressed nonviolent participants were matched on sex, relationship distress and age to distressed violent participants and reported only relationship distress and no physical aggression by either partner.

We also collapsed across gender in our experimental sessions. There are several studies that supported this decision. For example, over 200 studies have demonstrated at least gender symmetry in family-only IPV (53). Also, distressed violent females are as verbally aggressive as distressed violent males (25), and verbal aggression and physical aggression are highly correlated (54). Also, drinking alcohol within 3 h of an argument with a partner is a strong predictor of female IPV (55), and there are no gender differences in aggressive tendencies once males and females are drinking (56). There are also no gender differences among adults in the use of physical aggression once emotional arousal is present (57), nor gender differences in aggression under conditions of high provocation (58). Furthermore, follow-up analyses confirmed our assertion that there are no gender differences in physiological responding to the beverage condition (*F* = 0.710, *p* = 0.410) or stimuli (*F* = 1.278, *p* = 0.269) among distressed violent partners.

The partners selected for the experimental sessions returned to the laboratory on two separate occasions for counter-balanced alcohol and placebo beverage emotion-regulation sessions. For each session, participants were seated in a chair a comfortable distance from a TV monitor displaying stimuli, prepared for electrocardiogram recording, and then administered either an alcohol beverage or a placebo beverage. Participants engaged in a 5-min baseline Vanilla Task (59) while the recording of electrocardiogram (ECG) activity was collected. The Vanilla Task is a minimally demanding color detection task (viewing blocks as they change color and counting number of blue boxes) that has been shown to be superior to a resting baseline task in between- and within-baseline stability, amplitude and responsivity (60).

#### Emotion regulation task

The approach for studying emotion regulation in the present study has been used in several previous studies (61), but we utilized participant-tailored stimuli (video clips of respective partner’s evocative behavior) to enhance the emotional arousal, valence and salience the stimuli in the emotion regulation task. In the WATCH condition, participants were instructed to let their emotional experience occur naturally, and to pay attention to how they felt during the clip. In the DO NOT REACT condition, participants were instructed to attempt to suppress any feelings of emotion so as to prevent an observer from knowing that an emotional response had occurred. A total of 50 unique video clips between four and 8 s in length were used in the task; 25 evocative and 25 neutral. Each stimulus was presented twice: once in the WATCH condition and once in the DO NOT REACT condition. On each block of trials (WATCH or DO NOT REACT), participants viewed the instruction (WATCH or DO NOT REACT; 1.5 s), a blank screen (1 s), fixation cross (1.5 s), blank screen (0.5 s), video clip (4–8 s) and a blank screen (up to 2.5 s). The total amount of time required for the task was approximately 25 min.

#### Beverage protocol

##### Alcohol condition protocol

Participants received a mixed drink (cranberry juice and 100-proof vodka) intended to raise their blood alcohol concentration (BAC) to a target dose of 0.08 g% using a standard formula for calibrating alcohol doses to achieve target BACs. Specifically: Alcohol dose (g) = [(10 ^*^ BAC ^*^ TBW)/0.8] + [10 ^*^ MR ^*^ (DDP + TPB)] ^*^ [TBW/0.8; BAC = blood alcohol concentration, TBW = total body water, MR = alcohol metabolism rate, DDP = duration of drinking period, TPB = time to peak BAC; (57)]. Participants were asked to drink the beverage within 9 mins to ensure they remained on the ascending limb or reached peak BAC during the experimental task. Baseline recording began when participants reached a BAC of 0.06 g%.

##### Placebo condition protocol

Procedures were identical to the alcohol condition, except participants consumed a volume of juice equivalent to the volume of beverage consumed in the alcohol condition. To maintain blindness to the condition, the cup was misted with vodka and 3 milliliters of vodka was floated on top of the cranberry juice to produce the smell and taste of an alcohol beverage.

#### Heart rate variability recording and processing

Electrocardiogram (ECG) data were collected using the BrainVision actiCHamp 64-channel, DC amplifier, 24-bit resolution, biopotential system. Respiration and ECG were measured using an integrated BrainVision respiration belt, and an ECG in Lead II position. Baseline measures were collected, and ECG data were time locked to stimuli presentation during the emotion-regulation task. ECG data were quantified and measures of respiratory sinus arrythmia (RSA), a measure of parasympathic activity and cardiac vagal control (62), using the integrated QRSTool and CMetX to extract the inter-beat-interval (IBI) from the ECG data and calculate RSA from the IBI series (63).

## Results

Statistical Package for the Social Sciences 28 (SPSS 28) was used to perform the statistical analyses of the data for this study. Preliminary analyses were conducted to assure no violations of the assumptions of normality, linearity, multicollinearity and homoscedasticity. Because predictions for all analyses were directional, derived from theory and specified in advance, they were evaluated using a one-tailed criterion of significance (64). Assuming a two-tailed test, Type I error = 0.05, and *r* = 0.32 we conservatively projected that we would require 40 (20 DV, 20 DNV) participants to have sufficient statistical power (0.801) to reject the null interaction hypothesis. The study design is repeated measures within-subjects design with a between-subjects factor and the data analysis strategy utilized was a repeated measures analysis of variance with a between subjects factor (e.g., partner type) that follows directly from the study design. Significant main effects and interactions are reported below. A Bonferroni correction was used to correct for multiple comparisons. SPSS utilizes a mathematically equivalent adjustment and multiplies the observed (uncorrected) value of p by the number of comparisons made to obtain a corrected value of *p*. Please note that the corrected *p*-values are reported below.

### Effect of alcohol on respiratory sinus arrhythmia

**Figure 1:**
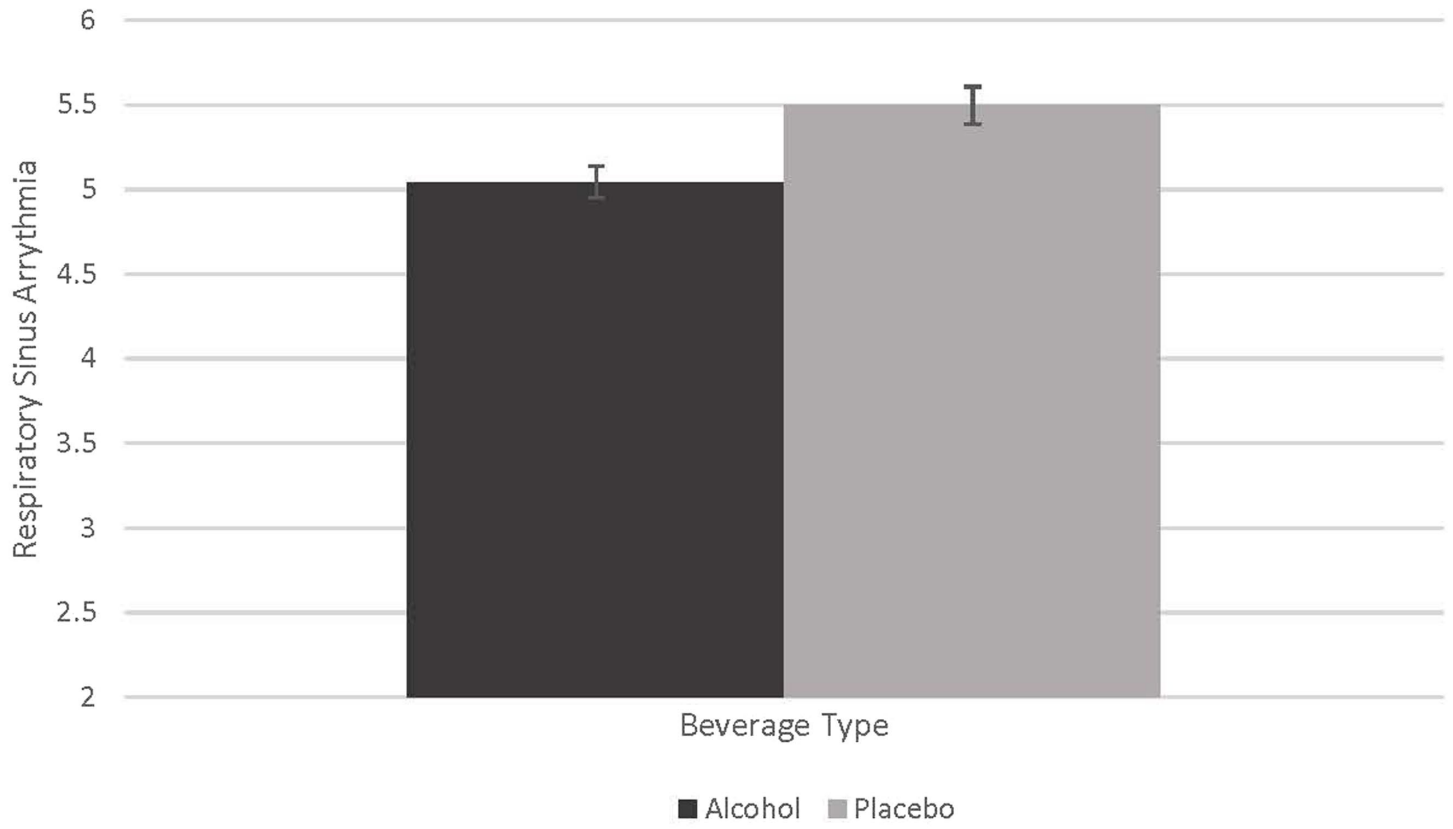
Effect of alcohol on respiratory sinus arrythmia. This figure demonstrates the marginally significant difference in beverage type on respiratory sinus arrythmia (RSA; *F* = 4.077, *p* = 0.51, partial eta squared = 0.102).

To examine the effect of alcohol on the respiratory sinus arrhythmia (RSA) measure of heart rate variability we conducted a repeated-measures analysis of variance (RMANOVA) and examined the main effect of beverage (Alcohol vs. Placebo) as the within-subjects factor collapsed across partner type. A Bonferroni correction was used to correct for multiple comparisons. The effect of alcohol on RSA was marginally significant (*F =* 4.077, *p* = 0.051, partial eta squared = 0.102). Although only marginally significant, alcohol produced lower RSA values than the placebo beverage. The effect size was large, however, suggesting an effect of alcohol on reducing RSA and thereby suggesting the capacity for effective emotion and behavior regulation is impaired by acute alcohol intoxication. See Figure 1.

### Distressed violent partners exhibited lower respiratory sinus arrhythmia when acutely intoxicated

**Figure 2:**
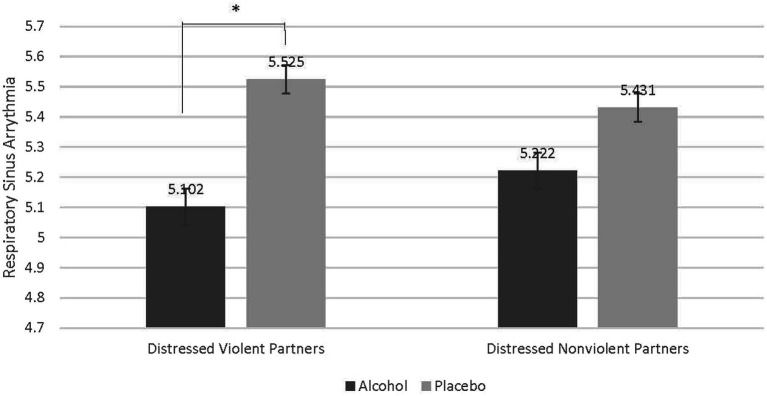
Contrast comparing decrease in respiratory sinus arrythmia in distressed violent partners when intoxicated. Compared to distressed nonviolent partners there was a statistically significant difference of the effect of alcohol on respiratory sinus arrythmia (RSA) in distressed violent partners (*M* difference = −0.424, *p* = 0.017). Acute alcohol intoxication reduced RSA in distressed violent partners to a greater degree than in distressed nonviolent partners.

There was a significant interaction between partner type (Distressed Violent vs. Distressed Nonviolent) and beverage type (Alcohol vs. Placebo; *F* = 6.300, *p* = 0.017, partial eta squared = 0.149) indicating that alcohol affected RSA differently in distressed violent and distressed nonviolent partners. To understand this interaction, contrasts compared alcohol to placebo beverage across distressed violent and distressed nonviolent partners. A Bonferroni correction was used to adjust for multiple comparisons. These contrasts reveal that compared to the placebo beverage, alcohol significantly reduced RSA in distressed violent partners, but not distressed nonviolent partners (*M* difference = −0.424, *p* = 0.017). See Figure 2.

### Distressed violent partners exhibit lower respiratory sinus arrhythmia when acutely intoxicated and attempting to regulate emotion

**Figure 3:**
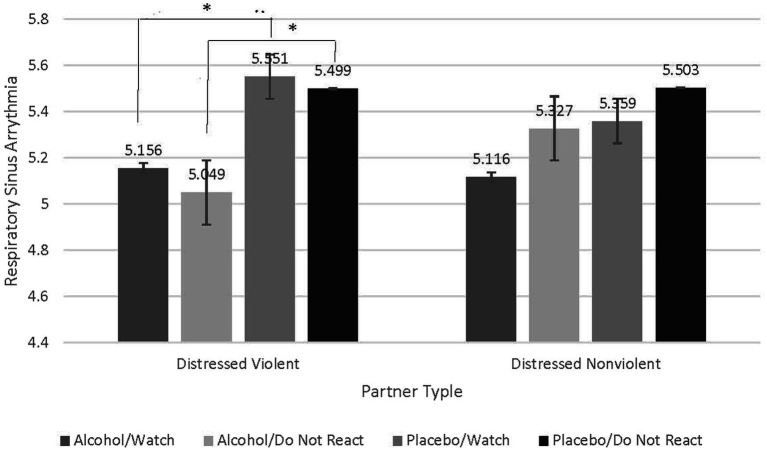
Contrasts examining significant interaction indicate greater decreases in respiratory sinus arrythmia in distressed violent partners compared to distressed nonviolent partners when intoxicated and attempting to not react to partner stimuli. Contrasts reveal that compared to distressed nonviolent partners, distressed violent partners, when intoxicated, exhibited significantly lower RSA when watching their partners’ stimuli (Watch; *M* difference = −0.395, *p* = 0.030). Distressed violent partners also exhibited even lower RSA when intoxicated and trying not to react to their partners’ stimuli (Do Not React; *M* difference = −0.450, *p* = 0.014). Distressed nonviolent partners did not exhibit significant differences in RSA in either emotion regulation condition under any beverage condition.

To test our hypothesis that distressed violent partners exhibit worse emotion regulation than distressed nonviolent partners as indexed by RSA, we conducted a repeated-measures analysis of variance (RMANOVA) and examined the interaction between emotion-regulation condition (Watch and Do Not React), beverage condition (Alcohol and Placebo), and partner type (Distressed Violent vs. Distressed Nonviolent). Results reveal a significant interaction (*F* = 5.092, *p = 0*.030, partial eta squared = 0.124). Follow-up contrasts were conducted to understand this interaction. A Bonferroni correction was used to adjust for multiple comparisons. Contrasts reveal that when intoxicated distressed violent partners exhibited significantly lower RSA when watching their partners’ stimuli (Watch; *M* difference = −0.395, *p* = 0.030) compared to intoxicated distressed nonviolent partners. Distressed violent partners also exhibited even lower RSA when intoxicated and trying not to react to their partners’ stimuli (Do Not React; *M* difference = − 450, *p* = 0.014). Distressed nonviolent partners did not exhibit significant differences in RSA in either emotion regulation condition while intoxicated. These findings suggest that not only do distressed violent partners exhibit worse capacity for emotion regulation, but this capacity is further worsened when attempting to not respond to their partners’ stimuli. See Figure 3.

### Distressed violent partners exhibit lower respiratory sinus Arrythmia when intoxicated and attempting to regulate emotion to evocative stimuli

**Figure 4:**
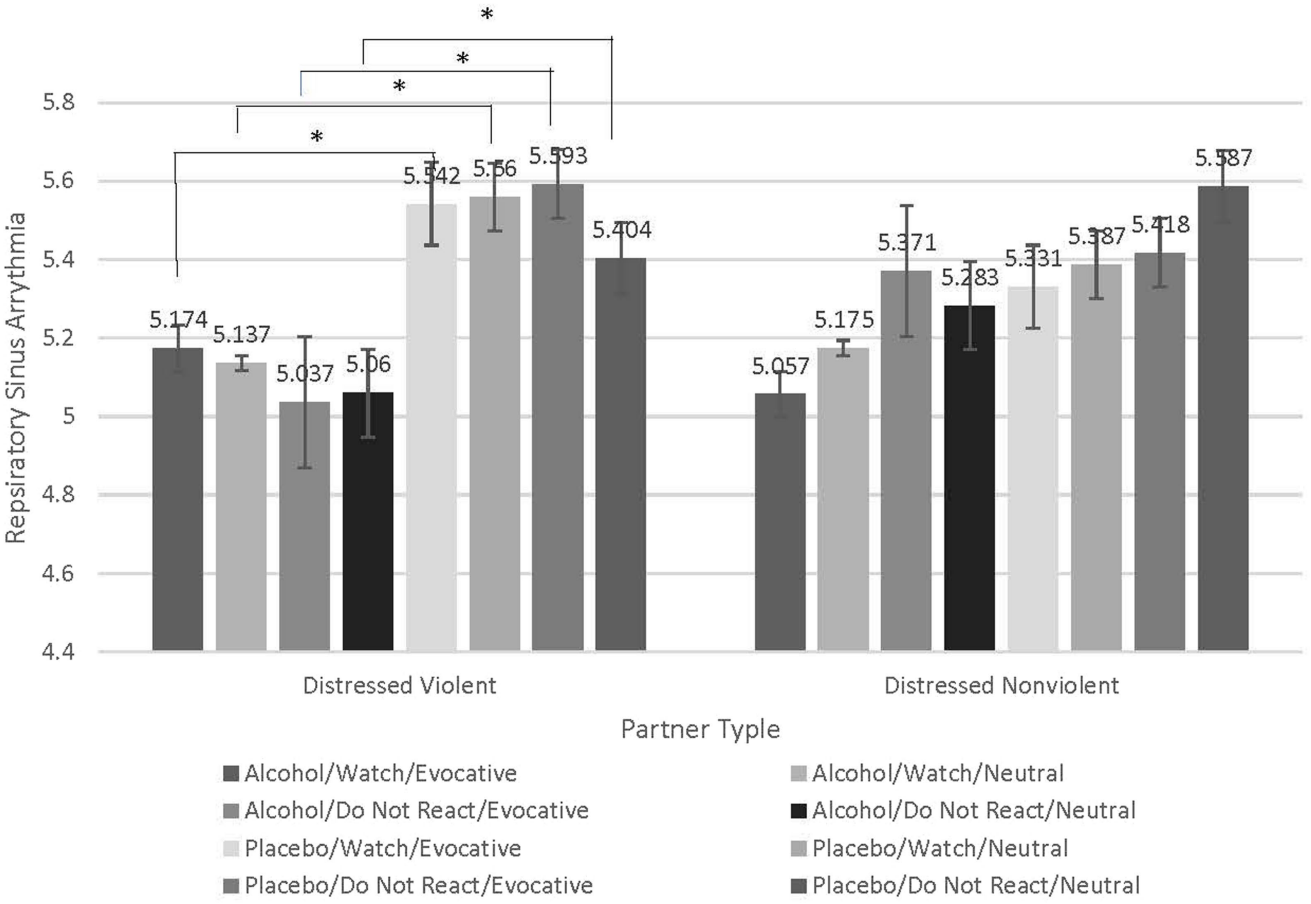
Contrasts examining significant interaction indicate greater respiratory sinus arrythmia decreases when distressed violent partners are intoxicated, attempting to regulate emotion in response to evocative partner stimuli. Contrasts conducted to understand the significant interaction between beverage condition (Alcohol vs. Placebo), emotion-regulation condition (Watch vs. Do Not React), stimuli type (Evocative vs. Neutral), and the between-subjects factor of partner type (Distressed Violent vs Distressed Nonviolent). Compared to distressed nonviolent partners, there were significant and large reductions in RSA when distressed violent partners were intoxicated and attempting to Not React to their partners’ evocative stimuli (*M* difference = −0.555, *p* = 0.009), and even when intoxicated and attempting to Not React to their partner’s Neutral stimuli (*M* difference = −0.345, *p* = 0.037). Although statistically significant, this effect to Neutral stimuli was not to the magnitude of that of the Evocative stimuli. Distressed violent partners also experienced a significant reduction in RSA when intoxicated and Watching neutral partner stimuli (*M* difference = −0.425, *p* = 0.019) which may have been an artifact of the pharmacological effects of alcohol. Distressed nonviolent partners did not exhibit significant differences in RSA in either emotion regulation condition under any beverage condition.

To test our hypothesis that distressed violent partners would exhibit reduced respiratory sinus arrythmia, thereby impaired emotional and behavioral control, compared to distressed nonviolent partners we conducted a repeated-measures analysis of variance (RMANOVA) and examined the interaction of beverage condition (Alcohol vs. Placebo), emotion-regulation condition [(Watch vs. Do Not React), stimuli type (Evocative vs. Neutral) within-subjects factors], and partner type (Distressed Violent vs. Distressed Nonviolent) as the between-subjects factor (See Figure 1). A Bonferroni correction was used to adjust for multiple comparisons. The expected beverage type by emotion-regulation condition by stimuli type by partner type interaction was statistically significant (*F* = 4.890, *p* = 0.033, partial eta squared = 0.120). To understand this interaction, we conducted follow-up contrasts which revealed counter-intuitive results. For distressed violent partners, there were no significant differences in RSA when Watching evocative partner stimuli in either beverage condition. Compared to distressed nonviolent partners there were, however, significant and large reductions in RSA when distressed violent partners were intoxicated and attempting to Not React to their partners’ evocative stimuli (*M* difference = − 0.555, *p* = 0.009), and even when intoxicated and attempting to Not React to Neutral partner stimuli (*M* difference *=* − 0.345, *p* = 0.037). Distressed violent partners also experienced a significant reduction in RSA when intoxicated and Watching neutral partner stimuli (*M* difference = − 0.425, *p* = 0.019) which may have been an artifact of the pharmacological effects of alcohol. None of the follow-up contrasts were significant for distressed nonviolent partners. These findings suggest that the method of emotion regulation that distressed violent partners adopt when attempting to not respond to their partners’ stimuli is maladaptive and worse when they are intoxicated, and their partners are behaving evocatively. See Figure 4.

## Discussion

The present study was an examination of the effects of acute alcohol intoxication on biobehavioral emotion regulation capabilities of distressed violent partners with our hypotheses being partially supported. We hypothesized that compared to distressed nonviolent partners, distressed violent partners experienced reduced respiratory sinus arrythmia, thereby reducing emotion regulation capabilities, under conditions of acute alcohol intoxication, viewing evocative partner stimuli and being asked to feel the emotions they associated with the evocative stimuli. While our hypotheses were supported with respect to partner type and the effects of acute alcohol intoxication, we found that distressed violent partners experienced reduced respiratory sinus arrythmia when acutely intoxicated and attempting to not respond to their partners’ evocative stimuli. This stands in contrast to distressed nonviolent partners who in this condition experienced increased respiratory sinus arrythmia. These findings suggest in the context of their severe relationship distress and acute alcohol intoxication, distressed violent partners may adopt strategies for emotion regulation that further impair their ability to respond adaptively. This finding stands in contrast to the distressed nonviolent partners who appeared to adopt a more adaptive emotion regulation strategy in this condition.

Rumination and suppression strategies are two emotion regulation strategies with particular relevance to partner violence. Watkins et al. (65) demonstrated that individuals produced more aggressive responses to provocation when asked to adopt a ruminative emotion regulation strategy when acutely intoxicated and believed to be in a competition with their partners. Rumination is thought to impel aggressive actions because it maintains a high level of physiological arousal, maintains focus on anger-inducing memories, and thoughts of retaliation (66). Similarly, experimental suppression techniques have been associated with reduced behavioral expressions of negative affect, but an increased experience of the negative affect and physiological activation (67), much like the responses of the distressed violent couples in our study. Suppression strategies have been shown to have many deleterious affective, cognitive and social consequences. Cognitively, these same techniques have also been shown to impair memory for details of conversations of interpersonal conflict (68). Socially, suppression techniques have been shown to cause greater stress in the partner of the individual exhibiting the suppression technique (69). Our previous research (52) demonstrated a very similar process in the partners in distressed violent participants. The partners of the distressed violent participants reported significantly greater effort in monitoring and preventing outward manifestations of anger, but that those attempts eventually failed and their own anger expressions took the form of contemptuous, critical and insulting comments and physically aggressive behavior. Further research is needed to fully elucidate this process, however.

We have also extended the findings from the physiological work with nonviolent couples in conflict (33) to distressed violent partners who, compared to distressed nonviolent partners, exhibited both overall sympathetic dominance as evidenced by the low respiratory sinus arrhythmia (RSA) measure of HRV, and even stronger sympathetic dominance when intoxicated, viewing evocative stimuli and attempting to regulate their emotional response. Such sympathetic dominance causes a loss of capacity to respond adaptively to introceptive (strong affective blends) and extroceptive (evocative partner behavior) stressors. Previous studies have shown low respiratory sinus arrythmia is associated with the loss of capacity to respond adaptively to introceptive and extroceptive stressors, as well as a tendency to respond to stressors with increased dysregulated affect (70–75).

Lastly, our choice of conducting a placebo-controlled alcohol administration study warrants further discussion. While some argue that alcohol administration studies should include a no-alcohol control conditions to control for compensatory behaviors often witnessed in placebo conditions (76), there were several factors that drove our decision to include only a placebo control condition in the present design. Our primary consideration was including a second control condition may cause participants to habituate to the effect of viewing the same evocative partner stimuli numerous times. This was an important consideration given that we employed a within-subjects design. With a no-alcohol control condition, participants would have been exposed to the same partner stimuli a total of six times, potentially reducing the stimuli’s evocative and physiologically arousing ability. In addition, such a procedure in the present study would have been unwieldy, a significant burden on participants (sessions were each 2 to 5 h long) and would have complicated the interpretation of an already complex set of findings. We also argue that a placebo condition versus a no-alcohol condition was the appropriate control condition in the present study. The effect of the substances often cannot be explained solely by their pharmacological properties, and expectations are partly responsible for how one responds to the effects of substances (77). This is particularly true for alcohol consumption where expectations may be learned through experiences and socialization, especially in the case of couple conflict. As such, placebo alcohol control conditions have been almost exclusively used to disentangle the effects of pharmacological and expectations on a range of behaviors, including aggression (78, 79). With the inclusion of a placebo beverage control condition, we felt we were best able to control for the expectancies surrounding alcohol use in couple conflict.

### Clinical implications

The present study is an analogue of costs to society from hazardous or harmful drinking that include putting individuals at a risk for violence. In addition, the largest proportion of individuals who report alcohol-related IPV do not report alcohol dependence symptoms. As such, this present study is representative of most alcohol-related IPV. Understanding the factors involved in alcohol-related IPV in this population is important for the development of treatments as current substance use treatment and conflict-focused couples treatments have proven to be insufficient to meaningfully influence the occurrence of alcohol-related IPV. This work has also identified novel targets for treating alcohol-related intimate partner violence. For example, in addition to behavioral treatments focused on improved emotion and behavior regulation, and conflict resolution in distressed violent couples (both partners), heart rate variability biofeedback (HRV-BFB) may enhance this learning and mitigate the loss of capacity to respond adaptively to interoceptive and exteroceptive stressors. HRV-BFB is an intervention delivered for disorders associated with affect dysregulation, including substance use disorders, PTSD, major depression, and anxiety disorders (70–75, 80). This biobehavioral intervention takes advantage of the respiratory sinus arrhythmia, that is, the innate entrainment of heart rate (HR) to the breath. Maximal increases in the amplitude of heart rate oscillation (i.e., higher levels of HRV) are produced when the cardiovascular system is rhythmically stimulated by paced breathing at a frequency of about 0.1 Hz [i.e., six breaths per minute; (81, 82)]. By instructing individuals in this specialized paced breathing technique using biofeedback visualization of their real-time respiratory and cardiac parameters, one can increase HRV (83), and at the same time increase sensitivity of the baroreflex, the body’s regulatory mechanism for dynamic control of HR and blood pressure (84). As a result, HRV-BFB can enhance parasympathetic nervous system functioning, autonomic stability and affect regulation (72, 84). It is important to note that a biofeedback procedure is necessary to accomplish this as one needs to learn to breathe at the resonant frequency of the cardiovascular system of each individual (85). This cannot be accomplished simply by relaxation or other deep breathing techniques.

Additionally, a key feature of the drinking rates of the participants in the present study was that we excluded individuals who showed signs of alcohol dependence. As such, participants exhibited, at worst, hazardous or harmful drinking levels. Decades of research has demonstrated that brief interventions for hazardous or harmful drinking are highly effective at reducing drinking to low risk levels (86–89). Recent reviews of IPV treatment also suggest promise for treatments that address substances (90). Given that the distressed violent couples in our study reported significantly more heavy drinking days than distressed nonviolent couples, providing a brief intervention to reduce their drinking to a low risk level, including not engaging in conflict with their partners when drinking, should also be a key feature of treatment.

## Limitations and future directions

There are several limitations of the present study which may limit the generalizability of our findings. First, we had unequal sample sizes in our two groups of partners. Recruitment of couples with conflict in their relationships yielded a largely distressed violent sample. In addition, most potential distressed nonviolent couples did not consume enough alcohol to qualify for an alcohol administration study. Future studies should attempt to over-recruit distressed nonviolent couples. Relatedly, these findings do not generalize to partners or couples with severe alcohol use disorders who would require treatment beyond a brief intervention to address the alcohol use disorder. Also, in an attempt to control for relationship stability, we recruited couples who were married or living together at least 6 months. This inclusion criteria may have been overly strict and not representative of couples who experience physical aggression in their relationships. Future studies should broaden the inclusion criteria to include couples who are also in dating relationships. Similarly, since this was the first of its kind investigation of heart rate variability in alcohol-related intimate partner violence our inclusion criteria were restricted to heterosexual couples. Future studies should extend these findings to same-sex couples to determine if similar processes are present.

## Data availability statement

The raw data supporting the conclusions of this article will be made available by the authors, without undue reservation.

## Ethics statement

The studies involving human participants were reviewed and approved by Human Research Review Committee, The University of New Mexico Health Sciences. The patients/participants provided their written informed consent to participate in this study.

## Author contributions

BF, EC, JC, and DH contributed to the design, the conduct of the study, and interpretation of study results. BF drafted the manuscript. EC, JC, DH, and JB provided critical feedback on the manuscript draft. All authors contributed to the article and approved the submitted version.

## Funding

This research was supported in part by the National Institute on Alcohol Abuse and Alcoholism (NIAAA) of the National Institutes of Health (AA022367) and the National Center for Advancing Translational Science (UL1TR001449 and KL2TR001448). The content is solely the responsibility of the authors and does not necessarily represent the official views of the National Institutes of Health.

## Conflict of interest

The authors declare that the research was conducted in the absence of any commercial or financial relationships that could be construed as a potential conflict of interest.

## Publisher’s note

All claims expressed in this article are solely those of the authors and do not necessarily represent those of their affiliated organizations, or those of the publisher, the editors and the reviewers. Any product that may be evaluated in this article, or claim that may be made by its manufacturer, is not guaranteed or endorsed by the publisher.

## Footnotes

^1^https://www.niaaa.nih.gov/Resources/ResearchResources/job22.htm
